# Supplementary material for: Prognostic value of composite inflammatory markers in patients with chronic obstructive pulmonary disease: A retrospective cohort study based on the MIMIC-IV database
Source: PLoS One. 2025 Jan 24;20(1):e0316390. doi: 10.1371/journal.pone.0316390 (PMC11761080; doi:10.1371/journal.pone.0316390)
Supplement: S2 File — (DOCX) [file pone.0316390.s002.docx]

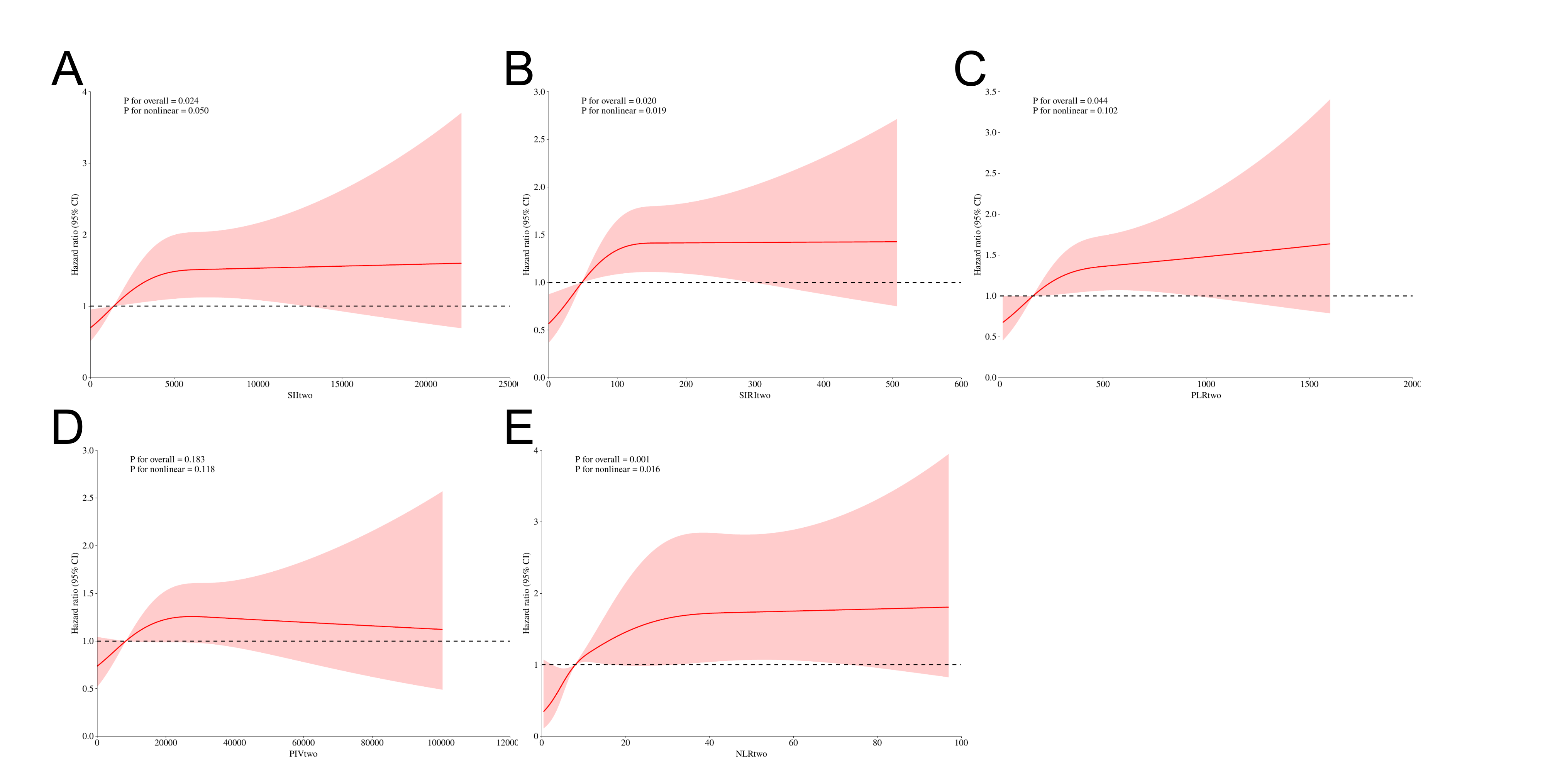


Fig S1. RCS of ICU mortality （A）SII；（B）SIRI；（C）PLR；（D）PIV；（E）NLR


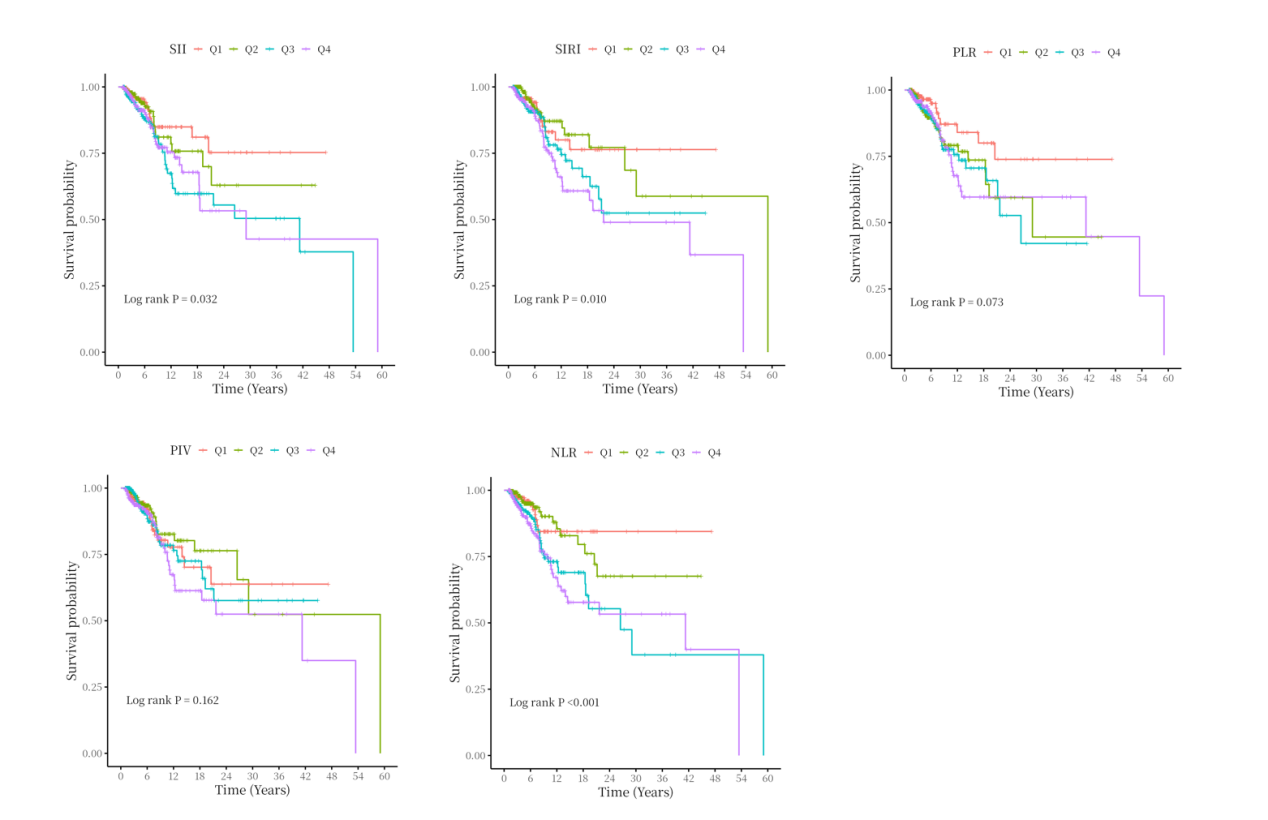


Fig S2. KM curve of ICU patients（A）SII；（B）SIRI；（C）PLR；（D）PIV；（E）NLR


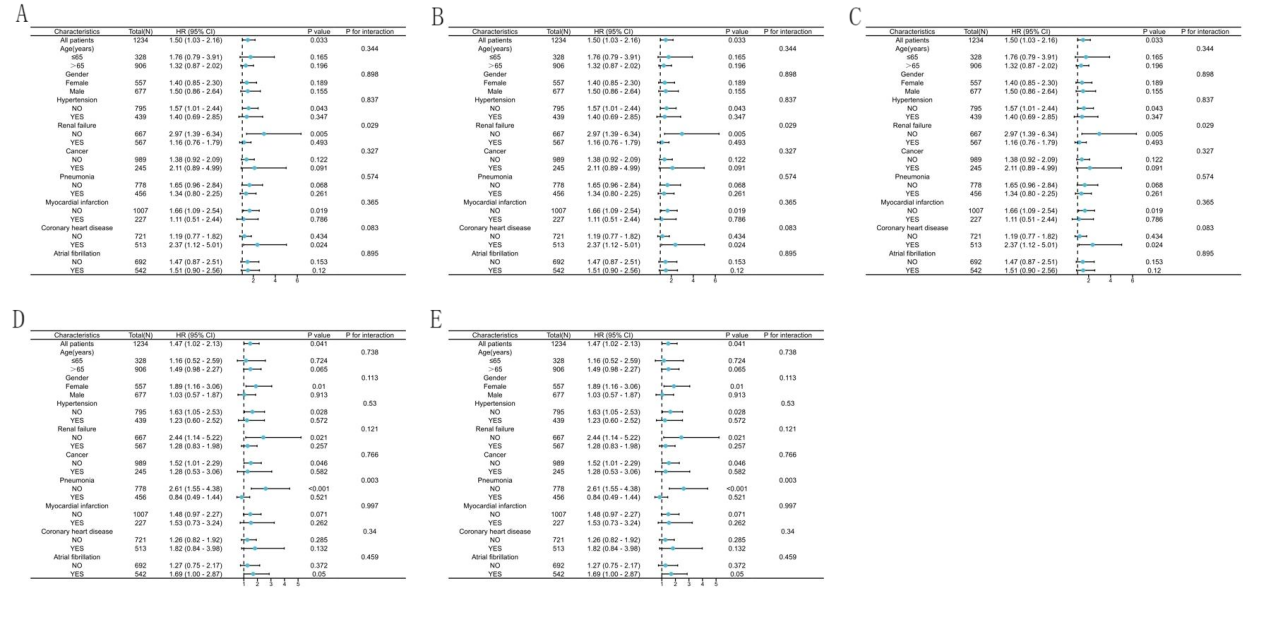


Fig S3. Subgroup analysis forest plot (ICU)（A）SII；（B）SIRI；（C）PLR；（D）PIV；（E）NLR.
